# Evaluation and comparison of a flumethrin-imidacloprid collar and repeated monthly treatments of fipronil/(s)-methoprene to control flea, *Ctenocephalides f. felis*, infestations on cats for eight months

**DOI:** 10.1186/s13071-016-1575-5

**Published:** 2016-05-17

**Authors:** Michael W. Dryden, Vicki Smith, Wendell L. Davis, Terry Settje, Joe Hostetler

**Affiliations:** Deptartment of Diagnostic Medicine/Pathobiology, Kansas State University, Manhattan, KS 66506 USA; Bayer HealthCare, LLC, Animal Health, P. O. Box 390, Shawnee Mission, KS 66201 USA

**Keywords:** *Ctenocephalides f. felis*, Cat flea, Imidacloprid, Flumethrin, Fipronil, (S)-methoprene, Collar, Cat, Feline

## Abstract

**Background:**

This controlled laboratory study was designed to evaluate the efficacy of the 10 % imidacloprid/4.5 % flumethrin collar (Seresto®, Bayer Animal Health) against fleas (*Ctenocephalides f. felis*) on cats, when compared to fipronil (9.8 %w/w)/(s)-methoprene (11.8 % w/w) topical spot-on formulation (Frontline® Plus for Cats and Kittens, Merial).

**Methods:**

Thirty cats were randomized into three groups of ten animals based on pre-treatment flea counts: Group 1: imidacloprid/flumethrin collar; Group 2: fipronil/(s)-methoprene topical spot-on and Group 3: non-treated controls. The imidacloprid/flumethrin collars were applied one time on Day 0, while the fipronil/(s)-methoprene spot-on was administered every 30 days from Day 0 through Day 210. Cats were infested with 100 fleas on study days 0, 7, 14, 29, 59, 89, 119, 149, 179, 209 and 239. All flea counts were conducted by combing to remove fleas on post-treatment days 2, 8, 15, 30, 60, 90, 120, 150, 180, 210 and 240.

**Results:**

The efficacy of the imidacloprid/flumethrin collar ranged from 98.2 to 100 % for eight months. The efficacy of fipronil/(s)-methoprene spot-on ranged from 68.2 to 99.9 %. Efficacy was < 85 % for fipronil/(s)-methoprene on Days 90, 150 and 210. The flea counts in both treatment groups were significantly fewer than those in the non-treated control group at every post-treatment study day (*P* < 0.0001). In addition, there were significantly fewer fleas in the imidacloprid/flumethrin collar group when compared to the fipronil/(s)-methoprene group on Days 90, 150 and 210 (*P* < 0.0001).

**Conclusions:**

This study demonstrated that the imidacloprid/flumethrin collar (Seresto®, Bayer Animal Health) maintained excellent ( > 98.2 %) efficacy against fleas on cats for the entire 8 month study. Monthly applications of fipronil/(s)-methoprene (Frontline® Plus for Cats and Kittens, Merial) generally had high, but variable (68.2 to 99.9 %) efficacy over the course of the eight month study. Based on the very high residual efficacy achieved by the imidacloprid/flumethrin collar in this study, veterinarians should expect that this collar will control and eliminate existing flea infestations on cats and in their in-home premises as long as every flea infested host is treated.

## Background

The cat flea, *Ctenocephalides felis felis* is an extremely important external parasite of dogs and cats. Infestations of pets and their surrounding premises can result in disease and great distress for pets and their owners [1]. Historically, numerous different insecticides and insect growth regulators have been used in a variety of formulations (shampoos, sprays, dips, foams, injectable, spot-ons, collars, powders and oral systemics) in attempts to control fleas on dogs and cats [1, 2].

The impregnated collar delivery system was once popular and widely used for flea control [1, 2]. Historically, a variety of insecticides such as carbaryl, chlorpyrifos, diazinon, dichlorvos, lindane, methylcarbamate, naled, propoxur, temophos and tetrachlorvinphos have been used in these collars [1–8]. While various levels of efficacy were clearly demonstrated in laboratory studies under controlled conditions [3–7], questions often persisted concerning their safety [9, 10] and their ability to manage an active flea infestation when subjected to continual challenge in a naturally flea-infested environment [11]. The inability of some of these collars to manage existing flea infestations and their poor management of flea allergy dermatitis may have been due to their slow residual speed of kill. Two studies demonstrated that chlorpyrifos and dichlorvos impregnated collars could not kill fleas fast enough to stop flea reproduction [3, 8]. If fleas are laying eggs, then they are living, feeding and injecting saliva for at least 24 h on the collared pet. Clearly, if fleas continue to reproduce on these collared pets then the infestation could be sustained. Based on perceived issues of efficacy and safety, use of insecticide impregnated flea collars declined precipitously in popularity with veterinarians.

More recently newer insecticide collar formulations have been introduced that contain either deltamethrin or imidacloprid-flumethrin [12–14]. These collars have a more prolonged activity against fleas and substantially higher levels of residual efficacy [12–14]. Imidacloprid and flumethrin are combined in a slow-release collar formulation for both dogs and cats containing 10 % imidacloprid (w/w) and 4.5 % flumethrin (w/w). It might be assumed that the imidacloprid in the collar is for the control of fleas, and the flumethrin is primarily for the control of ticks. However, it has been demonstrated in-vitro, using a Lepidoptera species, that there is synergistic effect of imidacloprid on the flumethrin [15]. This synergism may enhance the insecticidal properties of the collar. In a previous study, when the imidacloprid-flumethrin collars were placed on cats, flea control ranging from 89.1–95.5 % was maintained for at least eight months [13].

While these new collars appear to be a substantial advancement in formulation technology, topical spot-ons still are a widely used insecticide application mode for cats. One such product is a topical spot-on formulation of fipronil/(s)-methoprene. Several laboratory controlled studies have reported that the monthly residual efficacy of the topical spot-on formulation of fipronil/(s)-methoprene applied to cats against *C. f. felis* ranges from 71.4 to 100 % depending on study design and flea strain [16–19].

The purpose of this study was to compare and evaluate the initial and residual efficacy of the imidacloprid (10 % w/w)/flumethrin (4.5 % w/w) collar (Seresto®, Bayer Animal Health) and the topical spot-on formulation of fipronil (9.8 % w/w)/(s)-methoprene (11.8 % w/w) (Frontline® Plus for Cats and Kittens, Merial) against *C. f. felis* infestations on cats.

## Methods

### Animals

Cats were individually housed in cages for the duration of the study, they were let out of their cages daily to exercise. They were bathed with a mild, non-medicated shampoo, thoroughly combed, and allowed to acclimate for 11 days prior to the initiation of treatments. Concomitant treatments were prohibited except where deemed necessary and would not influence the performance of any product. Cats were included if they were > 6 months old, determined to be healthy based on physical examination, not pregnant, were able to harbour an adequate flea infestation, and were not exposed to a previous insecticide within 60 days of study onset. Initially, 36 purpose-breed Domestic Shorthair (DSH) cats were evaluated for inclusion in the study. One cat was removed on day −9 due to a pre-existing health issue. The 35 remaining cats were evaluated for the ability to harbor adequate flea infestations. Briefly, 35 cats were infested with approximately 100 non-fed, adult *C. f. felis* (Wildcat strain K-State) on day -6. On study day -5, flea counts were performed to remove live fleas. Cats were then ranked by pre-study flea counts in descending order and blocked in sets of 3. The 5 cats with the lowest flea counts were not included in the study. The remaining 30 cats were subsequently randomized by a pre-selected randomization schedule in an attempt to normalize the variability across treatment groups. Ten cats were allocated to each treatment group, to ensure that a minimum of 6 cats would be available for evaluation at each time point. All animal care procedures conformed to guidelines established by the Institutional Animal Care and Use Committee at Kansas State University (IACUC protocol No. 3181).

### Experimental infestations

Cats were infested with approximately 100 *C. f. felis* on study days 0, 7, 14, 29, 59, 89, 119, 149, 179, 209 and 239. Fleas used for all, but one infestation were from the KSU Wildcat strain. For the infestation on Day 209, due to a decrease in flea colony production, a different strain was used (BerTek). Flea counts were performed approximately 24 h following infestation except on Day 2 (48 h). The entire body of each cat was groomed with a single-sided, fine-tooth flea comb. Live fleas were collected, removed, frozen, and counted. Flea removal was achieved by combing each cat thoroughly for 10 min. If 5 or more fleas were recovered during this period, the cat was combed for an additional 5 min. If any fleas were recovered during the second combing, the cats were combed for an additional 5 min. Separate flea combs were used for each cat.

### Treatments

All products were applied in accordance with the manufacture’s guidelines and label recommendations. Cats were weighed and dosage adjusted accordingly. Cats in group 1 were treated by placing an imidacloprid (10 % w/w)/flumethrin (4.5 % w/w) (Seresto®, Bayer Animal Health) collar around their necks on day 0; the collars remained in place for the duration of the 240 day (8 month) study. Cats in group 2 were treated with a topical spot-on containing fipronil (9.8 % w/w)/(s)-methoprene (11.8 % w/w) (Frontline® Plus for Cats and Kittens, Merial) on Day 0 and then monthly through Day 210. Following the initial treatment on day 0, each of the 8 consecutive monthly treatments occurred immediately following that month’s flea removal. Cats in Group 3 served as non-treated controls.

### Clinical monitoring

The cats were observed at least once daily during the acclimation period and a physical examination was performed on each cat 10 days prior to starting the study to verify health status. On Day 0, cats were observed 1 and 4 h post application for adverse effects. For the remainder of the study, cats were observed daily until study completion or removal from the study at which time a physical examination was performed.

### Efficacy determination

Total body live flea counts were determined and recorded. Individual flea counts were used to calculate a geometric mean (GM) for each group on the specified study days. For each post-treatment flea count, efficacy was calculated using Abbott’s Formula. Percent efficacy (% reduction) was determined by comparing the GM number of live fleas retained on the treated group to the GM number of live fleas retained on the non-treated negative control group. For example: $\frac{\%\kern0.5em \mathrm{efficacy}}{\mathrm{Control}}=\frac{\mathrm{GM}\;\mathrm{flea}\ \mathrm{count}\ \left(\mathrm{control}\right)\hbox{-}\;\mathrm{G}\mathrm{M}\;\mathrm{flea}\;\mathrm{count}\;\left(\mathrm{treatment}\right)\times 100}{\mathrm{GM}\;\mathrm{flea}\;\mathrm{count}\ \left(\mathrm{control}\right)}$

### Data analysis

The assumption of equally distributed flea-ridding ability was assessed by descriptively summarizing the pre-study flea counts. The statistical method comparing post-treatment flea counts included the pre-study flea counts for each animal as the covariate. The efficacy of the treated groups, relative to the non-treated control group, was computed with Abbott’s formula. Geometric means were calculated following transformation using a logarithmic method (averaging the transformed values, and converting the average using antilog to represent a GM). Because some animals had zero flea counts, all counts were modified by adding one to each prior to logarithmic transformation. Also, one was subtracted from the antilog value to meaningfully represent the GM for each group. Log flea counts (flea count + 1) were analyzed with a repeated measures analysis of covariance (RMANCOVA) including terms for treatment (TRT), animal (random), study day (DAY), and the interaction of treatment and study day (TRT × DAY), using the pre-treatment flea counts as a covariate. SAS PROC MIXED Version 9.2 (SAS® Institute, Cary, NC) was used for analysis with the covariance structures ‘CSH’, for data collected on unequal intervals, which had the smallest Akaike Information Criterion. The interaction of treatment and study day was significant at the 0.05 level. The active treatment groups were compared to the control through the simple effect of TRT for each time point, with *P*-values adjusted for multiple group comparisons (Bonferroni). These simple effect pairwise comparisons were obtained from the TRT × DAY interaction.

## Results

The placement of a single imidacloprid/flumethrin collar (Seresto®, Bayer Animal Health) provided 98.2 to 100 % control of adult *C. f. felis* throughout the entire eight month study (Table 1). The eight monthly applications of fipronil/(s)-methoprene (Frontline® Plus for Cats and Kittens, Merial) resulted in efficacy ranging from 68.2 to 99.9 % (Table 1). Efficacy was < 85 % for fipronil/(s)-methoprene on Days 90, 150 and 210. The flea counts in both treatment groups were significantly fewer than those in the non-treated control group at every post-treatment study day (*t*_(251)_ < -5.45, *P* < 0.0001). In addition, there were significantly fewer fleas in the imidacloprid/flumethrin collar group when compared to the fipronil/(s)-methoprene group on Days 90, 150 and 210 (*t*_(251)_ < -3.10, *P* < 0.0001).

**Table 1:** Geometric (arithmetic) mean live flea counts and percent efficacy against the Wildcat strain of *C. f. felis* infesting cats treated with either an imidacloprid/flumethrin collar or a fipronil/(s)-methoprene topical spot-on

	Non-treated controls^d,g^	Imidacloprid/flumethrin^e,g^		Fipronil/(s)-methoprene^f,g^	
Study day	Mean No. of fleas	Mean No. of fleas	% control^h^ Geomeans	Mean No. of fleas	% control^h^ Geomeans
2	55.0^a^ (55.8)	0 (0)	100^b^	0.1 (0.1)	99.9^b^
8	55.3^a^ (57.9)	0 (0)	100^b^	0.6 (1.0)	98.8^b^
15	52.0^a^ (53.5)	0.9 (1.4)	98.3^b^	0.4 (0.7)	99.1^b^
30	47.0^a^ (48.7)	0 (0)	100^b^	0.2 (0.4)	99.5^b^
60	35.7^a^ (36.6)	0 (0)	100^b^	1.0 (2.5)	97.2^b^
90	40.4^a^ (42.4)	0 (0)	100^b^	12.8 (15.7)	68.2^c^
120	38.8^a^ (39.6)	0.1 (0.1)	99.8^b^	1.9 (4.0)	95.0^b^
150	45.9^a^ (47.3)	0 (0)	100^b^	7.1 (10.4)	84.5^c^
180	38.3^a^ (40.2)	0 (0)	100^b^	1.5 (2.6)	96.1^b^
210	46.8^a^ (47.3)	0.1 (0.1)	99.8^b^	12.5 (14.0)	73.3^c^
240	48.5^a^ (50.9)	0.9 (1.4)	98.2^b^	3.3 (4.1)	93.2^b^

^d^ Each of 10 cats in the control group received no treatment

^e^ Each of 10 cats in the imidacloprid/flumethrin group were fitted with a Seresto® collar (Bayer Animal Health) on day 0. Note that two cats were removed from this group (one on study day 2 and one on study day 10)

^f^ Each of ten cats in the fipronil/(s)-methoprene group were treated Frontline® Plus for Cats and Kittens (Merial) on day 0 and then monthly through day 210

^g^ Each cat was infested with 100 adult *C. f. felis* on days 0, 7, 14, 29, 59, 89, 119, 149, 179, 209, and 239

^h^ % efficacy/Control = (GM flea count (control) – GM flea count (treatment) × 100)/GM flea count (control) $\%\kern0.5em \mathrm{Control}=\frac{\mathrm{geometric}\;\mathrm{mean}\;\mathrm{count}\ \mathrm{control}\hbox{-} \mathrm{geometric}\ \mathrm{mean}\ \mathrm{count}\ \mathrm{treatment}}{\mathrm{geometric}\ \mathrm{mean}\ \mathrm{count}\ \mathrm{treatment}}\times 100$

^a,b,c^ Values across rows with unlike superscripts are significantly different (*P* < 0.0001)

### Clinical findings

Thirty cats, 17 males and 13 females, were included in the study. All cats were over 6 months of age with a mean weight of 3.5 kg (range 2.6–4.3 kg) 5 days prior to the start of the study. A number of adverse events were reported in all three treatment groups during the 8 month study. Adverse events reported in the imidacloprid/flumethrin collar group consisted of alopecia, erythema, abrasions, and single episodes of vomiting and diarrhea (unknown if product related). The alopecia could be attributed to the flea infestations and/or the mechanical irritation of the collar. However, the erythema and abrasions were attributed to the collars as the cats were not used to something around their neck. Of the adverse events in the fipronil/(s)-methoprene group, the alopecia and pruritus were attributed to the repeated flea infestations. However, one cat had alopecia at the site of product application and one cat developed diarrhoea. In the non-treated control group, there was a single report of vomiting and the other observations were alopecia and abrasions likely associated with repeated flea infestations.

Two cats in the imidacloprid/flumethrin collar group were removed from the study due to hair loss, erythema, and abrasions that developed underneath and next to the collar; one was removed on study day 2 and the other on study day 10. It appeared as though the cats were licking or scratching at these areas and increasing the severity of the lesions.

## Discussion

The imidacloprid/flumethrin collar eliminated every flea (100 % efficacy) within 48 h of collars being applied to cats. This clearly demonstrates a rapid distribution of insecticide from the collar. This collar also provided ≥ 98.2 % control of flea infestations within 24 h of each re-infestation for at least 8 months. In fact, the efficacy was 100 % within 24 h following five of the eight monthly re-infestations. The 8 month residual efficacy of the imidacloprid/flumethrin collar observed in this study was similar too or higher than the previous reported efficacy range of this collar on cats of 89.1–95.5 % [13].

Historically, insecticide impregnated flea collars likely failed to control flea infestations because their residual speed of flea kill was too slow to prevent fleas from producing viable eggs [3, 8, 11]. *Ctenocephalides f. felis* can initiate egg production within 24–48 h after females acquire a host and take their first blood meal [20, 21]. Therefore, if a residual insecticide cannot kill newly acquired fleas within 24 h, egg production could be maintained and the infestation will be sustained generation upon generation. However, if the newly acquired fleas can be killed prior to initiating reproduction, then the infestation on the cat and in the surrounding premises will be eliminated because if a population cannot reproduce it will be eradicated in the local in-home environment [22, 23]. Based on the very high 24-h residual efficacy achieved by the imidacloprid/flumethrin collar in this study, this collar should be able to control and eliminate existing flea infestations on cats and in their in-home premises as long as every flea infested host is treated.

The fipronil/(s)-methoprene topical spot-on formulation was generally efficacious against the *C. f. felis* strains used in this study. Efficacy was 99.9 % within 48 h of application and the 30 day residual was > 93.2 % following five of the eight monthly applications. However, there were three times that the 30 day residual efficacy dropped below 85 %. This occurred on days 90, 150 and 210. It is unknown why the efficacy was statistically lower following these infestations. While the BerTek strain was used for the day 209 infestation with a resulting 73.3 % efficacy for fipronil/(s)-methoprene, following the day 90 infestation with the Wildcat strain, the efficacy of the spot-on formulation was 68.2 %. Therefore, it does not appear that a different flea strain can account for the reduced efficacy.

Another observation from the data is that it does not appear that any significant bioaccumulation of fipronil occurred following 8 consecutive monthly applications. Efficacy 30 days following the eighth application was 93.2 % while the 30 day residual efficacy following the first application was 99.5 %.

Based on post-launch pharmacovigilance data, and other reports in the literature, the erythema, alopecia, and abrasions that developed underneath and next to the collars in several of the cats in this study appears to be an uncommon occurrence. A laboratory study that evaluated *Bartonella henselae* transmission to cats found that 0/8 cats treated with the imidacloprid/flumethrin collar developed lesions associated with the collar [24]. A clinical field study, that evaluated the safety and efficacy of the imidacloprid/flumethrin collar (*n* = 232) in cats naturally infested with fleas and/or ticks revealed that 9.0 % of the imidacloprid/flumethrin treated feline study population developed lesions associated with the collar compared to 5.6 % of the control population (n = 81); the difference was not statistically significant (*P* > 0.05) [25]. Similar results were observed in a second field comparison that evaluated 95 cats owned by veterinary students or teaching hospital staff and treated with either the imidacloprid/flumethrin collar or had a placebo collar applied. All dermal lesions were observed during the first 14 days of wearing the collar and none of these lesions were significantly different between groups. Multivariate analysis revealed that young cats that had not previously worn collars were significant covariates. The authors concluded that cats that were originally intolerant of collars became more receptive over time and the adverse events observed in imidacloprid/flumethrin treated cat were similar to cats wearing a placebo collar and also to cats wearing an identification collar reported in a previous study [26].

Therefore, the aforementioned studies suggest that the adverse event results reported here are not consistent with findings reported by other investigators or pharmaco-vigilance data reported by Bayer Animal Health (per communication with Drs. Cristiano Von Simson and Joe Hostetler). The degree of collar associated lesions observed in this study may be a reflection of cage confined cats that were not used to wearing a collar and were “worrying” at the new device. However, the results should not be discredited and owners should observe their cats occasionally, especially during the first few days of product application, to ensure the collar is not placed too tightly and that lesions do not develop.

## Conclusions

This study confirmed that the imidacloprid/flumethrin collar maintains excellent efficacy (98.2–100 %) against fleas on cats for at least eight months. In addition, the imidacloprid/flumethrin collar performed favourably to the fipronil/(s)-methoprene topical spot-on, and the imidacloprid/flumethrin collar group had significantly fewer fleas when compared to the fipronil/(s)-methoprene group on Days 90, 150 and 210. Based on the very high 24-h residual efficacy achieved by the imidacloprid/flumethrin collar in this study, this collar should be able to control and eliminate existing flea infestation on cats and in their in-home premises as long as every flea infested host is treated.

### Ethics statement

The present research was approved and complied with the regulations set forth by the Kansas State University Institutional Animal Care and Use Committee. This research protocol IACUC # 3181 approved 31July 2012.
